# Water pollution drives environmental degradation in a seasonally influenced Neotropical coastal river

**DOI:** 10.1007/s10661-025-14956-w

**Published:** 2026-03-16

**Authors:** Leonesa da S. Belanha, Otacílio L. S. Paz, Andre A. Padial, Júlio C. R. de Azevedo, Robert M. Hughes, Renata Ruaro

**Affiliations:** 1 Federal Technological University of Paraná, Graduate Program in Environmental Science and Technology, Curitiba, Paraná, Brazil; 2 Laboratory of Biomonitoring and Applied Ecology, Department of Chemistry and Biology. , Rua Deputado Heitor Alencar Furtado, Curitiba, PR 5000, 81280-340 Brazil; 3 State University of Paraná, Campus União da Vitória, União da Vitória, PR Brazil; 4 Laboratory of Geoprocessing and Environmental Studies, Federal University of Paraná, Curitiba, Paraná, Brazil; 5 Laboratory of Analysis and Synthesis in Biodiversity, Botany Department, Federal University of Paraná. Av. Cel Francisco H Dos Santos, S/N, Curitiba, PR 81513-000 Brazil; 6 Amnis Opes Institute, Glenn, Corvallis, OR 2895 SE USA; 7 Department of Fisheries, Wildlife, & Conservation Sciences, 104 Nash Hall, Corvallis, OR USA

**Keywords:** Anthropogenic stressors, Guaraguaçu River, Invasive species, Multiple stressors, Tropical coastal river

## Abstract

Aquatic and coastal ecosystems are increasingly altered by human activities, particularly through the removal of riparian vegetation, pollution, urbanization, and biological invasions. This study aimed to assess environmental conditions and identify a gradient of human disturbance in the Guaraguaçu River, located on the coastal plain of southern Brazil and subjected to multiple stressors. To achieve this, we evaluated land use, water and sediment quality, and the dominance of invasive macrophytes, and applied a rapid physical habitat assessment protocol quarterly throughout 2024 at 22 sites along the main channel of the Guaraguaçu River and its two tributaries, the Pery and Pombas Rivers. Data were summarized using principal coordinate analysis (PCoA), and the axis scores were used to define the environmental gradient. The results revealed a clear gradient in environmental conditions, likely driven by anthropogenic pressures across the watershed. Among the various stressors analyzed, water pollution emerged as the primary driver of environmental degradation in the Guaraguaçu River Basin. Conductivity, salinity, ammonia, and orthophosphate contributed most significantly to the observed gradient. The highest values for these parameters were recorded in the Pery River and downstream from its confluence with the Guaraguaçu River, indicating a decline in environmental quality in this tributary and, consequently, in the lower basin sector. The results showed that environmental conditions varied along a gradient significantly influenced by both seasonality and river zone, suggesting that seasonal dynamics are a key driver of this pattern. This influence of seasonality was also supported by the PCoA results, which reflected clear temporal patterns in environmental variability. Furthermore, human-induced changes to the Pery River, particularly water pollution and hydrological modifications, have impaired ecological conditions and intensified environmental degradation in downstream areas of the Guaraguaçu River basin. These findings highlight the need for targeted management strategies, including improved wastewater treatment, to mitigate cumulative stressors and enhance the resilience of this coastal river ecosystem.

**Supplementary Information:**

The online version contains supplementary material available at 10.1007/s10661-025-14956-w.

## Introduction

Freshwater ecosystems are stressed by catchment land use changes, riparian deforestation, pollution, flow modification, overexploitation, biological invasions, and the climate crisis (Dudgeon et al., 2006; Kefford et al., 2023; Reid et al., 2019). These stressors alter river flows, thermal regimes, and in-stream habitats and increase nutrient, sediment, and contaminant levels, resulting in deterioration of ecological conditions and ecosystem health (Dalla-Corte et al., 2020; Karr et al., 2022; Fanelli et al., 2022). In coastal river systems, ecosystem dynamics are shaped by a combination of factors, including changes in land use and land cover, variations in precipitation, sea level rise, and saltwater intrusion (Herbert et al., 2015; Osland et al., 2016; Tully et al., 2019). On coastal plains, wetland degradation and low slopes hamper drainage during heavy rainfall, extending the flooding duration in these areas (Silva et al., 2008). Urbanization within basins further degrades hydrological regimes through the expansion of impervious surfaces, installation of drainage infrastructure, and watercourse channelization, all of which reduce infiltration, alter water retention, and modify flow patterns (Marçal & Lima, 2016; Shaikh et al., 2023). Moreover, the introduction of invasive macrophytes poses additional threats to native biodiversity (Clavero & García-Berthou, 2005; Guerin et al., 2018) and habitat structure (Galvanese et al., 2022; Macêdo et al., 2024), contributing to sedimentation and flow disruption (Leonard et al., 2002; Temmerman et al., 2005; Yang, 1998; Zuo et al., 2025). For instance, invasive macrophytes often form dense mats that reduce light availability and alter nutrient cycling, which affects habitat complexity and leads to a decline in native biodiversity (Sato et al., 2021; Bora & Padial, 2023). Their dense growth can also impede water flow, promoting sediment accumulation and further modifying benthic habitats (Strayer, 2010) .

Because of the unprecedented pressures on freshwater ecosystems and the rapid decline in freshwater biodiversity (He et al., 2019; Reid et al., 2019), scientists have emphasized the need for scaling up efforts and prioritizing efficient management and conservation of these ecosystems to slow, halt, or reverse these trends (Albert et al., 2021; Maasri et al., 2022). In response to the ongoing global environmental crisis, the United Nations (UN) declared the “Decade of Ocean Science for Sustainable Development” which began in 2021 (Lopes & Rotta, 2022). This initiative aims to raise awareness and conservation about ocean and coastal basin pollution and the preservation of marine resources (Lopes & Rotta, 2022). To achieve that goal, monitoring, assessing, and mapping the ecological conditions of coastal basins are crucial because monitoring helps identify the most altered areas and the key stressors contributing to adverse effects (Allan et al., 2023; Herlihy et al., 2020). Monitoring also highlights regions with low human impact, pointing to potential opportunities for conservation (Allan et al., 2023; Fanelli et al., 2022).

In Brazil, national legislation regulates the water quality standards for surface waters. CONAMA Resolution No. 357/2005 (National Environmental Council) establishes limits for physical (e.g., temperature, conductivity, turbidity), chemical (e.g., pH, dissolved oxygen, nitrate), and biological (e.g., total coliforms, thermotolerant coliforms) variables to be assessed in monitoring programs. These limits are associated with the different classes of river conditions. Although this resolution remains Brazil’s principal monitoring framework, it presents key limitations. For instance, its guidelines are inspired by international frameworks and lack proper consideration of Brazil’s diverse ecological contexts (Passos et al., 2018). For effective assessment of freshwater conditions, it is essential to incorporate an analysis of basin landscape and environmental conditions (Rosenfield & Müller, 2020). However, many monitoring programs rely solely on physical and chemical water assessments to gauge environmental quality (Pati et al., 2023), which limit the data interpretation and hinder informed decision-making for environmental management (Simmons et al., 2021). Several tools are currently available for comprehensive environmental monitoring of river basins (Tsatsaris et al., 2021). Rapid assessment protocols (RAPs) (Cionek et al., 2024), indicators derived from Geographic Information Systems (GIS) (Tsatsaris et al., 2021), and water and sediment quality are commonly used approaches (Bownik & Wlodkowic, 2021; Gutiérrez et al., 2004).

Rapid assessment protocols provide a framework for evaluating environmental conditions using easily accessible information from the river or stream channel and surrounding area. They characterize physical habitat structure because river physical structure affects the biodiversity and the types of organisms present in a given habitat (Barbour et al., 1999; Cionek et al., 2024), as do sediment and water quality (Gutiérrez et al., 2004; Nascimento et al., 2019). The combination of land use, physical habitat, and water quality variables enhances the evaluation of environmental conditions in areas with varying land uses, helping to identify key factors that contribute to either good or poor conditions (Zhang et al., 2023a, 2023b).

The coastal region of Paraná holds significant national and international economic importance because of the establishment of services and infrastructure that support global markets, mainly related to industrial port services for agribusiness and the oil industry (Sezerino & Tiepolo, 2016). The Guaraguaçu River is a lowland coastal river in the Lagamar region of Paraná state, Brazil, within the Atlantic Rainforest biome (Reis et al., 2015; Vitule, 2008). The Lagamar region, a network of estuaries in south and southeast Brazil, is one of the best-preserved regions in the biome. It holds immense conservation value and is recognized as both a Biosphere Reserve and a World Heritage Site (Galvanese et al., 2022). The river’s drainage flows northward toward Paranaguá Bay, creating a vast floodplain surrounded by the marine coastal plain (Elste et al., 2019). This basin provides essential ecosystem services such as fishing, navigation, water supply, and recreational activities (Faria et al., 2021). It also supports various endemic fish species (Abilhoa & Duboc, 2004). However, the Guaraguaçu River basin faces land use pressures including rising water demand, expansion of port activities, rapid population growth, high levels of untreated sewage, and landfill leachates (Marques, 2017). Additionally, the river is threatened by invasive non-native species (Bora et al., 2020; Faria et al., 2021) and macro-drainage projects that introduce large volumes of saltwater into freshwater reaches (Mamede et al., 2015). The Guaraguaçu River mouth is situated near coastal areas that attract a high concentration of tourists during the summer, which contribute to urbanization in the catchment area (Torres & Tiepolo, 2022). A previous study by Elste et al. (2019) reported increased pollution, removal of riparian vegetation, erosion, and Pery River channel alterations. The river channels, originally created to drain land intended for resort construction, have undergone siltation and eutrophication. In recent years, agricultural land use in the Guaraguaçu Basin has increased. Notably, in 2017 alone, agricultural land expanded by 9.91%, altering both mountainous and lowland vegetation (Torres, 2019).

Therefore, we conducted an integrated environmental assessment to evaluate the gradient of environmental conditions, as well as spatial patterns and temporal dynamics, across the lower Guaraguaçu River system. This approach combined the application of a rapid assessment protocol (RAP), analyses of water and sediment quality, the dominance of invasive macrophytes, and land use characterization. Specifically, sediment quality indicators included the proportion of fine sand, coarse sand, and silt, which can reflect depositional processes. Regarding land use and land cover, we considered anthropogenic activities such as agriculture, pasture, and mining. We evaluated the hypothesis that the interaction between land use and land cover, water pollution, and dominance of invasive macrophytes determines an environmental degradation gradient in the Guaraguaçu River basin, and that this gradient is modulated by seasonality. Detecting this gradient is crucial for supporting effective management and conservation strategies in estuarine and riverine environments.

## Material and methods

### Study area

**Fig. 1:**
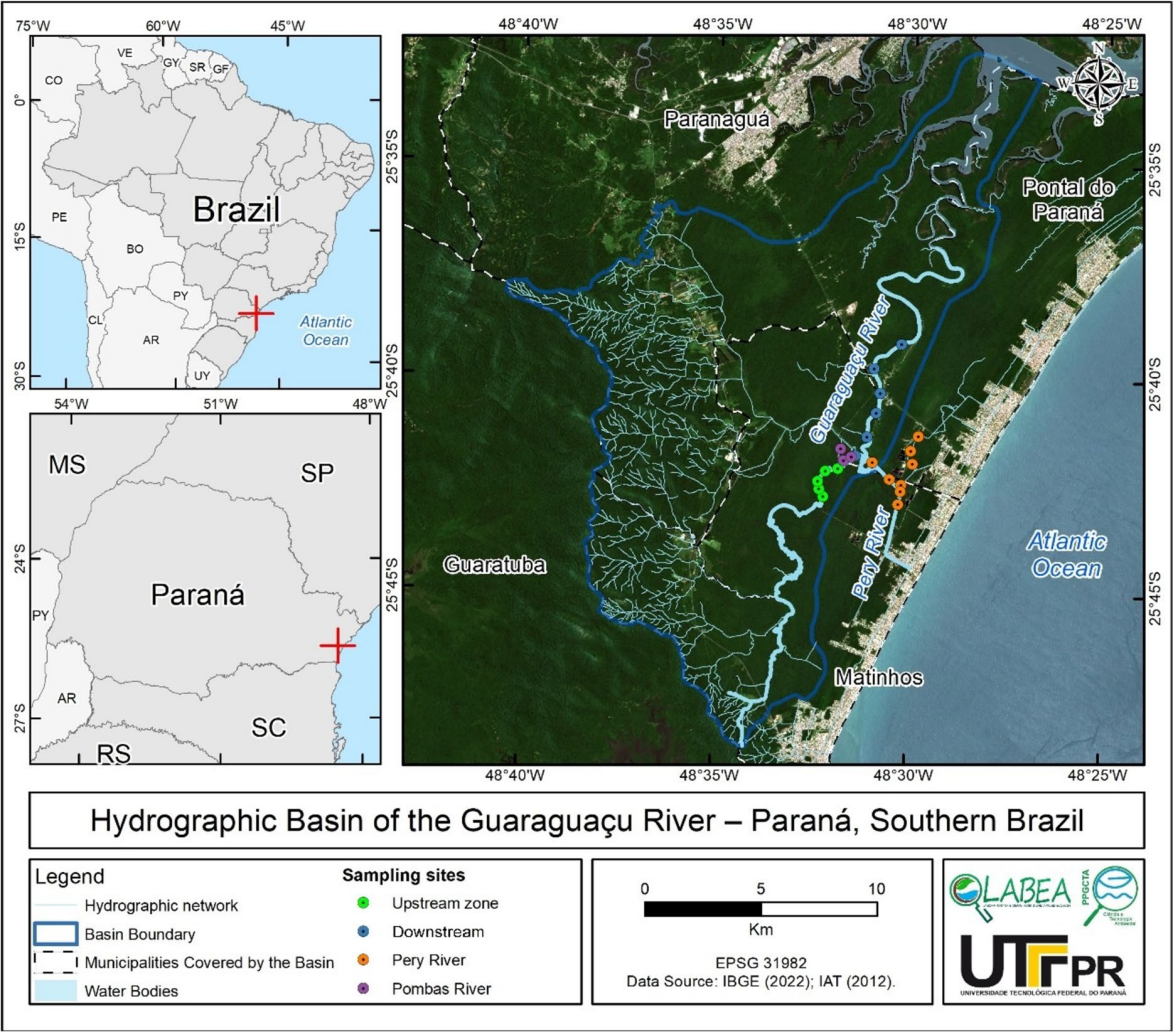
Study area and site locations. The Guaraguaçu River flows from south to north

The Guaraguaçu River Basin (BHRG) is part of the Coastal Hydrographic Sub-Basin of the South Atlantic Hydrographic Region (Fig. 1). The study area is in the central portion of that region and includes mountains and plains (Bigarella, 2009). The basin features diverse vegetation, including Dense Ombrophilous in Lowland, Submontane, Montane, and Upper Montane zones, as well as Pioneer Formations influenced by fluvial-marine (mangroves), marine (sandbanks), and fluvial (swamps and caixetais) environments in the lowlands (Roderjan et al., 2002). The climate is humid subtropical (Abilhoa & Duboc, 2004), with an average annual precipitation of 2300 mm (Vanhoni & Mendonça, 2008). Rainfall is consistent year-round but varies significantly between summer and winter. Winter is the driest season, with around 280 mm of rain, whereas summer is the wettest, with approximately 800 mm of rain (Vanhoni & Mendonça, 2008).

The Guaraguaçu River is characterized by a salinity gradient heavily influenced by tidal fluctuations (Elste et al., 2019). Additionally, there is a notable longitudinal variation, with distinct microhabitats along the river’s course (Lana et al., 2001). The river supports a diverse range of habitats, from wetland ecosystems to mangroves (Faria et al., 2021). Sites G1–G5 are in a nearly pristine area, whereas sites G6–G11 have degraded water quality (Fig. 1). Given the variations in anthropogenic pressures and environmental characteristics along the river, it can be divided into four distinct zones. (1) A nearly pristine upstream zone (sites G1, G2, G3, G4, G5; Fig. 1; Table S1, A1) features a “caixetal” ecosystem—characterized by an abundance of the endangered *caixeta* tree (*Tabebuia cassinoides*) (Araújo et al., 2021). (2) An intermediate zone is marked by water quality degradation from human occupation and brackish water (sites G6, G7, G8, G9, G10, G11; Fig. 1). In the intermediate region of the Guaraguaçu River, there are two straightened canals used for public water supply and domestic wastewater disposal. This area is also home to invasive macrophyte species, such as the tropical tanner grass *Urochloa arrecta* [(Hack. ex T. Durand & Schinz) Morrone & Zuloaga] and the water hyacinth *Pontederia crassipes* Mart. *P. crassipes* can become invasive in Brazilian waters despite being native (Michelan et al., 2013; Pavão et al., 2017). This invasive species is absent in the upstream portion of the river, as well as in much of the mangrove swamp near the river mouth (Bora et al., 2020). The final zone approaches an estuarine environment, where tidal influences further shape river characteristics (Araújo et al., 2021). (3) The Pery River (sites PER1, PER2, PER3, PER4, PER5, and PER6) was straightened and connected to a drainage system of artificial channels. These channels drain Pontal do Paraná and Matinhos municipalities (sites POJ and POM), receive sewage effluents and leachate from the Pontal do Paraná landfill, and drain areas lacking sewage treatment (Elste et al., 2019). In contrast, (4) the Pombas River tributary (sites POO1, POO2, and POR) is in a more pristine region within a mosaic of Conservation Units, including Saint-Hilaire/Lange National Park, Palmito State Park, and the Pombas River Ecological Station. This river serves as a water supply for municipalities in the region.

### Sampling

We sampled quarterly in March, June, September, and December 2024 at 22 sites, classified by region: Main channel (G1, G2, G3, G4, G5, G6, G7, G8, G9, G10, G11), Pery River (PER1, PER2, PER3, PER4, PER5, PER6, POJ, POM), and Pombas River (POO1, POO2, POR; Table S1). All sites were sampled within the same week of each sampling month. These months were selected to represent different hydrological periods in the region, including the end of the rainy season (March), the transition to the dry season (June), the dry season (September), and the return of rains (December). This temporal design aimed to capture seasonal variability in environmental conditions. Each site was represented by 50 m around the georeferenced site to balance the need to cover the environmental variability of the river section with the operational feasibility of sampling multiple sites (Downes et al., 2002). All sites were sampled in each campaign, allowing the use of repeated measures across time in the multivariate analyses. This approach helped evaluate both spatial patterns and temporal dynamics across the river system. To support the interpretation of seasonal environmental variability, accumulated precipitation data during the 2024 sampling period were compiled from AGUASPARANÁ – Paraná Water Institute, Hydrological Information System (available at http://www.sih-web.aguasparana.pr.gov.br/) and are presented in Table S5.

### Land use assessment

To assess land use and land cover around the sampling sites, we used geographical information system (GIS) tools. A 100-m-radius buffer was created around each sampling site using the “Buffer” tool in QGIS version 3.36.1-Maidenhead (QGIS.org, 2024). A vector dataset of Paraná coast vegetation, mapped at a 1:5000 scale between 2020 and 2021 (Britez, 2023), was applied. This dataset was cross-referenced with the buffers using an intersection operation. The original land use and land cover mapping was obtained through visual interpretation of satellite imagery from 2021, available in the Google Earth Pro collection. The original classification followed the guidelines of the Brazilian Vegetation Technical Manual (IBGE, 2012) and the Land Use Technical Manual (IBGE, 2013). Visual interpretation involved assessing colors, textures, structures, and the spatial position of landscape features, supported by geospatial data such as the hydrographic network, contour lines, and soil maps. The screen scale was set at 1:2500, and the minimum mappable unit was 0.05 ha (500 m^2^). The original vector data classes were later reclassified into three main categories: native vegetation, urbanized areas, and agricultural/pasture areas. This classification reflects an integrated land use and land cover approach, as the mapped categories represent both the physical characteristics of the surface (land cover) and their anthropogenic function (land use), thereby combining the material and functional dimensions of the landscape.

### Rapid Assessment Protocol (RAP)

The RAP used to assess environmental conditions in this study has 14 parameters and was adapted from Callisto et al. (2002) (Table S2a, b). To apply this protocol, we excluded vegetation cover on the riverbed, water transparency, oiliness of the bottom, extent and frequency of rapids, substrate types, mud deposition, sedimentary deposits, and water flow characteristics. We added siltation, riparian land use (main activity), erosion and/or stability of river banks, siltation, anthropogenic disturbances, water odor, oiliness of water, odor of sediment, riverbed type, channel changes, presence of native riparian vegetation, extent of riparian forest, presence of aquatic plants, presence of invasive aquatic plants, and presence of aquatic life (Table S2a, b). All parameters were assessed visually.

The parameters were categorized into three or four groups based on physical habitat quality, reflecting a gradual decline in habitat complexity. Each category was scored from 0 to 9 based on observations at the sampling site (Table S2a, b). The scores were summed to provide a quantitative assessment of the ecological conditions. After assigning values to each parameter, the total score for each site was calculated to determine site disturbance level (Cionek et al., 2011, 2024). The sum of the scores for all parameters reached a maximum of 126, offering an overall physical quality assessment (Barbour et al., 1999). To classify the total RAP values, the scores were entered into the attribute table of a vector file corresponding to the sampling points, using QGIS version 3.36.1-Maidenhead (QGIS.org, 2024). The resulting classifications were based on the sum of the scores obtained in the 14 parameters of the adapted RAP, resulting in three categories: natural areas (91–104 points) present well-preserved riparian vegetation, stable banks, absence of signs of pollution, and a significant presence of aquatic fauna; preserved areas (61–90 points), although exhibiting moderate alterations, they still maintain relevant ecological characteristics, such as significant vegetation cover and relatively intact ecosystem functions; altered areas (52–60 points) show evidence of environmental degradation, such as loss of vegetation, intense erosion on the banks, presence of solid waste, and low biological diversity. We considered natural vegetation the plant cover that occurs along the banks and adjacent areas of the waterbody without direct or significant interference from recent human activities. This vegetation includes restinga formations, mangroves, dense rainforest (ombrophilous forests), wetlands, riparian forests, and other plant community’s characteristic of the coastal zone, composed of arborous, shrubby, and herbaceous species forming a multi-strata vegetation (Cionek et al., 2024).

### Limnological variables

We assessed several abiotic variables, including physical and chemical properties of water, sediment granulometry, and organic matter content in the sediment. Key water variables, such as pH, temperature, dissolved oxygen (DO), conductivity, salinity, oxidation–reduction potential (Pot.Redox), total dissolved solids (TDS), and oxygen saturation (OS), were assessed on site using a portable Hanna HI9829 multiparameter device. For additional variables like nitrite (N-nitrite), nitrate (N-nitrate), ammoniacal nitrogen (N-ammonia), total phosphorus (total-P), and orthophosphate (ortho-P), water samples were collected and sent to the Laboratory of Advanced Studies in Water Chemistry (LEAQUA) at the Federal Technological University of Paraná (UTFPR), following the methodology described in Standard Methods for the Examination of Water and Wastewater (APHA, 2012).

Nitrite (N-nitrite), ammonia-N, nitrate (N-nitrate), and orthophosphate (ortho-P) analyses were performed on filtered samples (0.45-µm cellulose acetate membrane). Ammonia-N was determined by the Phenate/Indophenol blue method (4500-NH_3_ F). Nitrite-N was quantified through its reaction, in acidic medium, with sulfanilamide solution and naphthyl-1-ethylenediamine dihydrochloride (4500-NO_2_ B). Nitrate–N was quantified after reducing nitrate to nitrite in a cadmium column and then quantified through the reaction of nitrite, in acidic medium, with sulfanilamide solution and naphthyl-1-ethylenediamine dihydrochloride (4500-NO_2_ B). Orthophosphate was determined through the ammonium molybdate and potassium antimony tartrate method (4500-P E). Total phosphorus was quantified with strong acid, as described in APHA (2012), via ammonium molybdate and potassium antimony tartrate method (4500-P E). Water transparency (Secchi depth (cm)) was assessed with a Secchi disk.

We collected sediment by using a Petersen grab sampler with a sampling area of approximately 0.0189 m^2^. At each site, a composite sample was obtained from three subsamples. The granulometric composition of the sediment was analyzed according to Suguio (1973). Grain classification was carried out based on the Wentworth scale (Wentworth, 1922). To assess organic matter content, sediment samples were heated in a muffle furnace at 550 °C for 4 h to induce mass loss by incineration. The organic matter content was calculated as the difference between the mass of the oven-dried soil and the residue left after incineration (Teixeira et al., 2017).

We also gathered data on the dominance of the invasive species *U. arrecta* at each sampling site to evaluate biological invasion as a potential stress factor along the environmental gradient. At each sampling site, the abundances of *U. arrecta* and other aquatic plants were visually estimated using Braun-Blanquet classes (Mareel, 1975). A site was considered dominated by the invasive plant if the abundance of *U. arrecta* exceeded that of all other plants.

### Determination of the gradient of environmental conditions and data analysis

To evaluate the combined impact of land use, water pollution, physical habitat degradation, and biological invasion, data on RAP, land use, limnological variables, and the dominance of invasive aquatic plants were integrated to assess the environmental gradient of the Guaraguaçu River. Before combining the data, transformations were applied to minimize the impact of extreme values. Continuous positive data were transformed using the natural log (or ln + 1 for the variable that had zero values), data with both positive and negative values were transformed using the cubic root, and percentage data were transformed using the arcsine of the square root (Johnson & Wichern, 2002). pH is already on a logarithmic scale, so values were not further transformed. After applying transformations to the other variables when needed, all variables, including pH, were standardized by their standard deviation to ensure equal contribution to the statistical analysis. A Euclidean distance matrix was then created to calculate the distances between the sites. Principal coordinate analysis (PCoA, Gower, 1966) was applied, with the “Lingoes” correction to adjust for negative eigenvalues, allowing for a Euclidean space representation of the relationships between sites based on the combined environmental variables. A correlation analysis between the standardized variables and the PCoA axes was used to identify the key variables influencing each principal coordinate.

Permutational multivariate analysis of variance (Permanova; Anderson, 2001) was used to assess whether the environmental variables distance matrix could be explained by descriptor variables, including river zone, river type (main channel and tributary), dominance of invasive *U. arrecta* (Table S3), and season, as well as the interaction between season and those variables. We assessed five zones: (i) Guaraguaçu upstream (Upst), sites located upstream of the Pery River tributary; (ii) Guaraguaçu downstream (Down), sites located downstream of the Pery River tributary; (iii) Pery: sites on the Pery River; (iv) Pombas, sites on the Pombas River; and (v) wastewater disposal (Wast), sites near the wastewater treatment plant. Regarding the dominance of the invasive *U. arrecta*, the term “yes” indicates that *U. arrecta* occupied more than 50% of the macrophyte cover at the site. The total *R*^2^ value of the model was used as an indicator of how well it explained the environmental variables; significance was considered when type I error was lower than 5%.

Analyses were conducted in R 4.4.1 (R Core Team, 2024) using the “vegan” package (Oksanen et al., 2015) for Permanova, data standardization, and distance matrices; “dplyr” (Wickham et al., 2020) for data manipulation and transformation; “ape” (Paradis, 2012) for PCoA; and “ggplot2” (Wickham, 2011) for generating graphs.

## Results and discussion

### Land use

**Fig. 2:**
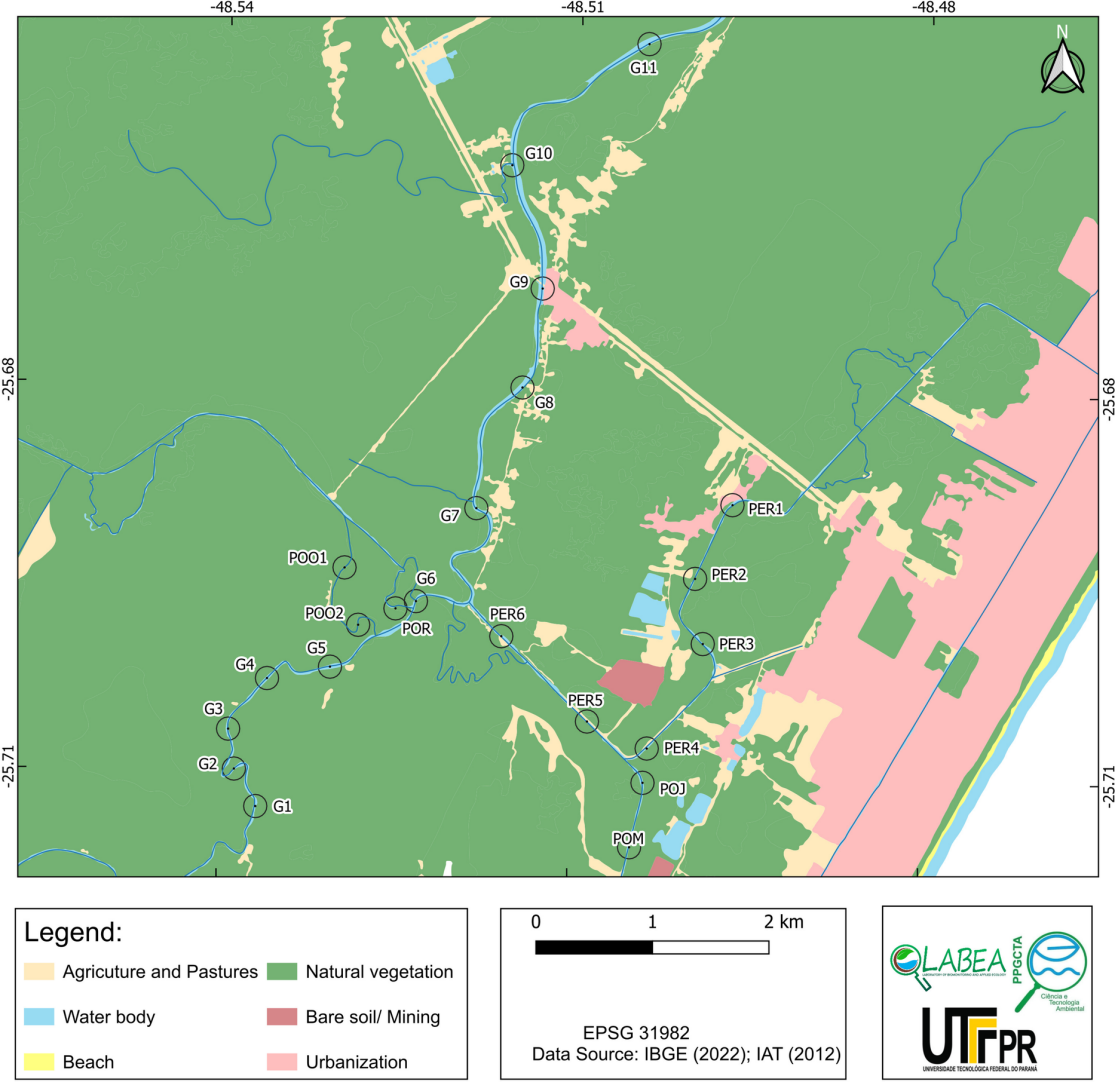
Land use and land cover map of the study area in the Guaraguaçu River basin, Brazil, showing sampling sites along the river system

The land use surrounding the study sites included agriculture and pasture, urbanization, and bare soil/mining; however, natural vegetation was predominant at most sites (Fig. 2). Specifically, at sites G1, G2, G3, G4, G6, POO1, POO2, POR, POM, and POJ, only natural vegetation was present within the 100-m buffers. At sites G5, G7, G8, G10, PER2, PER3, PER4, PER5, and PER6, both agricultural areas and pastures were found alongside natural vegetation. Sites G9 and PER1 featured a mix of agricultural areas, pastures, urban development, and natural vegetation.

Areas dominated by natural vegetation (G1, G2, G3, G4, G6, POO1, POO2, POR, POM, and POJ) exhibited land use with minimal human activities, conducive to environmental conservation (Britez, 2023). In contrast, sites characterized by natural vegetation, agriculture, and pasture (G5, G7, G8, G10, PER2, PER3, PER4, PER5, and PER6) indicated a transitional land use pattern likely altering site ecosystem condition (Torres, 2019; Santana & Araújo, 2021). Sites with a mix of urban development (G9 and PER1) experienced greater anthropogenic pressure, leading to vegetation fragmentation and likely reduced biological carrying capacity (Reinert et al., 2007; Rojas et al., 2021). These patterns of land use underscore the importance of integrated management and conservation strategies to balance human activities with the preservation of natural resources in the study area (Vieira et al., 2022).

### Rapid Assessment Protocol (RAP)

Based on the RAP scores, 12 of the 22 sites were classified as natural, eight sites as preserved, and two sites (G9 and PER1) as altered. Sites upstream of the Pery River confluence were classified as natural, whereas none of the sites located in the Pery River was natural (Fig S1). The natural RAP scores were obtained primarily in the areas farthest from human activity, particularly in the upstream sections (G1–G5), such as Rio das Pombas, and at several sites downstream (G7, G10, G11). The visual RAP highlighted notable changes in environmental quality, especially in the Pery River, a region most affected by anthropogenic influence (Torres, 2019).

The RAP has provided a valuable interpretation of the conservation status of water bodies elsewhere because of its flexibility (Machado et al., 2015). Visual assessments are recognized as effective descriptors, particularly in the evaluation, monitoring, and diagnosis of the physical and environmental quality of areas or rivers affected by human disturbances (Doll et al., 2016; Guimarães et al., 2017; Hughes et al., 2010). Additionally, these assessments assist in identifying the availability and quality of physical habitats for aquatic fauna in rivers (Bentos et al., 2018).

These results have provided important insights for the adaptive management of coastal basins by highlighting how different land use classifications (natural, preserved, and altered) have exhibited contrasting patterns of water quality and ecological dynamics over time (Torres, 2019). Identifying specific temporal variations between these categories allows managers and policymakers to target conservation and restoration efforts more effectively, prioritizing more vulnerable areas and adjusting strategies based on observed seasonal and hydrological conditions (Wang et al., 2023). For example, maintaining and restoring areas with preserved native vegetation, especially riparian forests, is crucial to mitigating the impacts of pollution and preserving the aquatic ecosystems (Wang et al., 2023).

### Water and sediment quality and environmental condition gradient

The Pery River and lower Guaraguaçu sites exhibited average dissolved oxygen concentrations below the recommended threshold of 5 mg/L (Brasil, 2005; Table S4). Reduced dissolved oxygen levels typically reflect elevated biochemical oxygen demand, associated with the decomposition of organic matter from anthropogenic or natural inputs (Genovez et al., 2024; Soares et al., 2023). These hypoxic conditions adversely affect aquatic biota (Bulbul Ali & Mishra, 2022), promoting shifts in community structure by favoring tolerant taxa while excluding more sensitive species (Liang et al., 2024). Based on the classification criteria established by CONAMA Resolution No. 357/2005 (Brasil, 2005), water bodies without an officially approved classification are, by default, considered Class 2. For the Guaraguaçu River, there is currently no formal classification in force for the entire river course. Instead, the State Environmental Institute (IAT) proposes a classification (enquadramento) in its technical document, suggesting that stretches of the Guaraguaçu River be designated as Class 2 (CERH-PR, 2020). In this context, the river is therefore treated as a Class 2 water body, which supports multiple beneficial uses, including potable water supply after conventional treatment, protection of aquatic life, recreational activities, irrigation, aquaculture, and fishing (Svolenski, 2000). However, the persistently low dissolved oxygen concentrations indicate the need for enhanced solid waste management, the establishment or expansion of domestic sewage collection systems, and improvements in the efficiency of wastewater treatment processes prior to effluent discharge into the river.

The low concentration of dissolved oxygen concentrations in certain parts of the river may be linked to increased nutrient inputs and higher concentrations of suspended or dissolved solids in the water. Furthermore, low oxygen concentrations can trigger anaerobic processes, which generate toxic byproducts such as sulfides and ammonia, further worsening water quality and harming aquatic fauna (Ursenbacher et al., 2020). In environments characterized by high conductivity, excessive nutrient availability can lead to algal blooms, which subsequently increase biological oxygen demand and lead to oxygen depletion (Esteves, 1998; Smith & Schindler, 2009). These changes in water quality variables, particularly turbidity and conductivity, are often associated with elevated levels of nutrients and organic matter (Dos Santos et al., 2024; Mangan et al., 2020). High concentrations of suspended or dissolved particles can significantly alter the ecological balance of aquatic systems, potentially impairing important ecological processes such as photosynthesis (Esteves, 1998; Lin et al., 2024) and affecting biotic and abiotic components of the aquatic ecosystem.

Regarding sediment assessment, fine sand predominated at most sampling sites (*n* = 16), accounting for over 40% of the sediment composition (Table S4). Larger particles, such as pebbles, were absent at all sites. This pattern is typical of areas with soils rich in fine materials, such as floodplains, where such sediments tend to accumulate (Zhang et al., 2023a, 2023b).

**Fig. 3:**
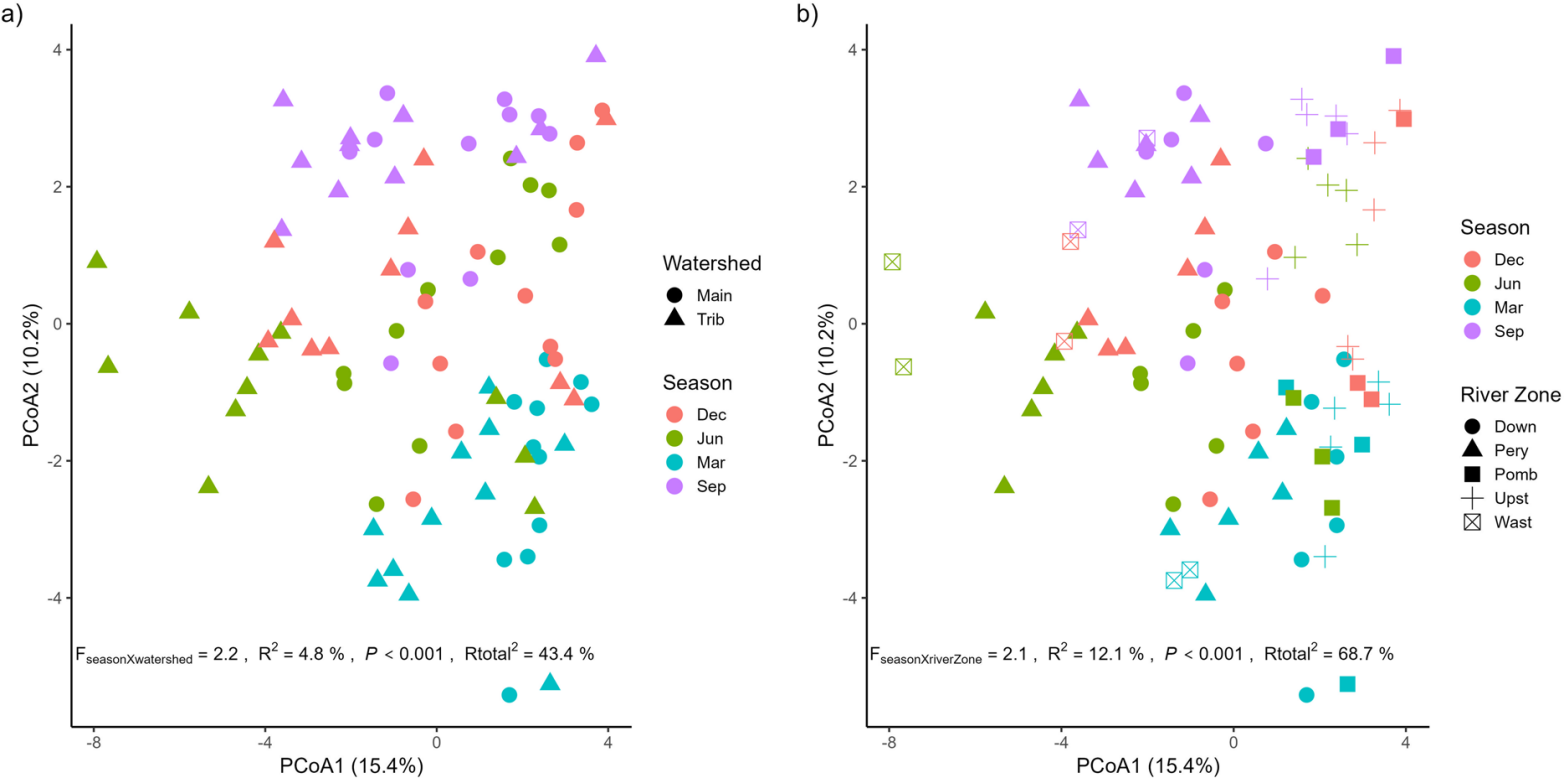
Principal coordinate analysis (PCoA) highlighting the differences between **a** river region (main channel and tributary) and sampling seasons (based on the month when all samples were collected within the same week) and **b** sampling seasons and river zone. The Permanova results of the significant interactions are also shown, in addition to the total explanatory coefficient of the models. Main = main channel of the Guaraguaçu River; Trib = Tributary of the Guaraguaçu River; Mar = March/2024; Jun = June/2024; Sep = September/2024; Dec = December/2024; Down = downstream; Pery = Pery River; Pomb = Pombas River; Upst = Upstream; Wast = wastewater disposal region

**Fig. 4:**
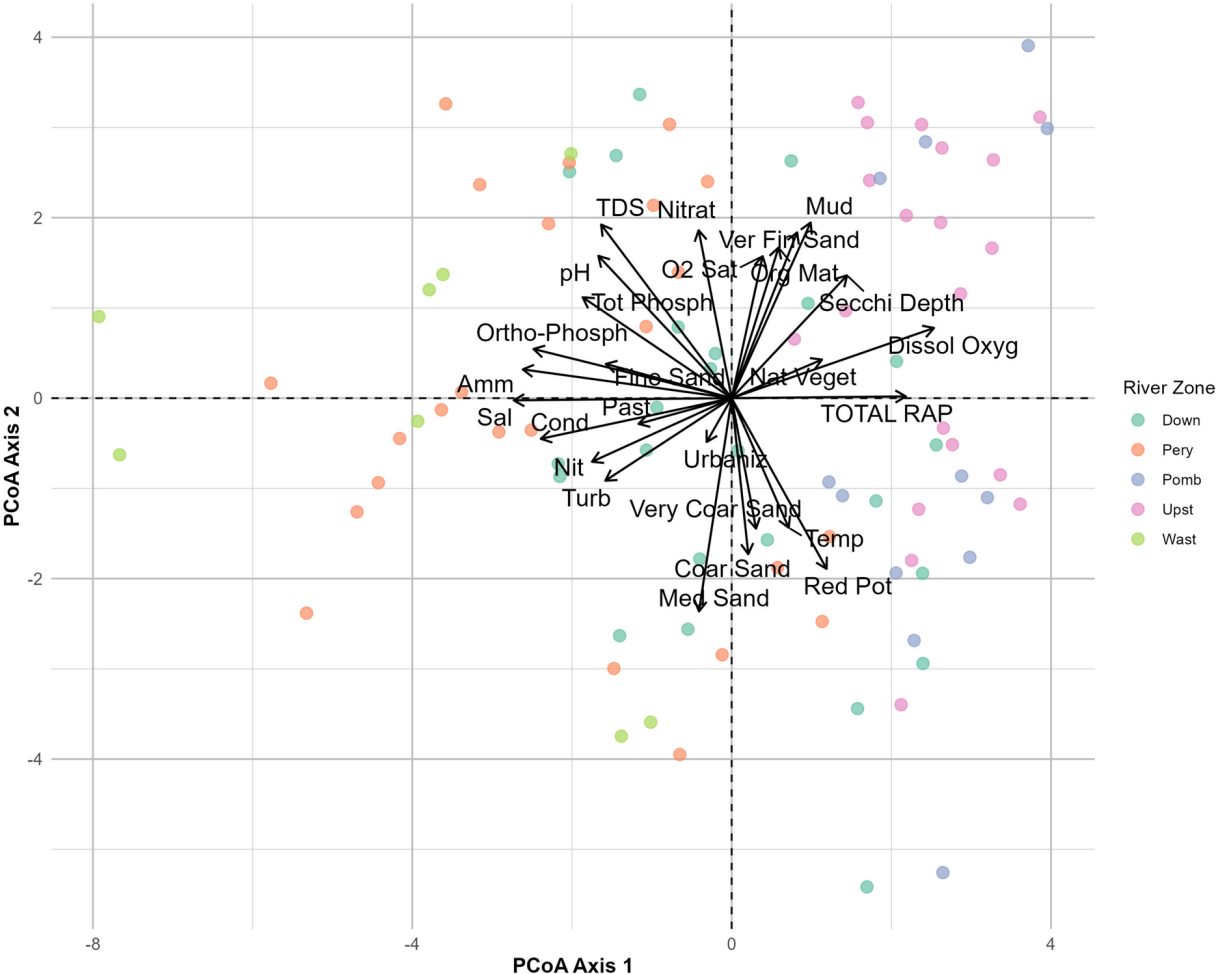
Principal coordinate analysis (PCoA) biplot showing the distribution of samples collected in different river zones (Down, Pery, Pomb, Upst, Wast) represented by different colors. The vectors indicate the environmental variables that most influence the variation between samples, including water physicochemical parameters (e.g., salinity, conductivity, dissolved oxygen), nutrients (nitrate, ammonia, total phosphorus), sediment characteristics (mud, fine sand, coarse sand), and factors related to land use (urbanization, natural vegetation). The separation of samples along axes 1 and 2 highlights significant environmental differences between river zones. Axis 1 reflects a physical and chemical gradient, while Axis 2 represents variation mainly associated with sediment and nutrient conditions. Down = downstream; Pery = Pery River; Pomb = Pombas River; Upst = Upstream; Wast = wastewater disposal region

There were clear seasonal differences between the main river channel and its tributaries (*F*_SeasonXWatershed_ = 2.2; *R*^2^ = 0.05; *P* < 0.001; *R*_total_^2^ = 0.43, Fig. 3a, Fig. 4). Tributaries can introduce large volumes of water, nutrients, and contaminants into the main channel, altering its environmental conditions (Carling et al., 2010; Konagai & Sattar, 2012; Savi et al., 2020; Shi et al., 2014).

Seasonality shaped ecological conditions by directly influencing zonal abiotic variables (*F*_Season X River Zone_ = 2.1; *R*^2^ = 0.12; *P* < 0.001; *R*_total_^2^ = 0.69, Fig. 3b; Fig. 4). These changes appear to be associated with changes in precipitation (Table S5), affecting nutrient concentration, salinity, and turbidity (Choi et al., 2021; Mullen et al., 2023; Yuan et al., 2023). For instance, in June and September (Table S5), reduced precipitation likely reduced the effluent dilution capacity, resulting in a higher concentration of pollutants in areas more susceptible to anthropogenic influence (Sampaio et al., 2023). Furthermore, in zones nearer to the mouth, reduced river discharge likely contributed to increased salinity because of reduced seawater dilution (Roy & Zahid, 2021). This variation also may have been related to tidal influences, especially near the mouth, which affect circulation and saline intrusion (Sant’Ana & Cunha, 2022). This natural dynamic, however, can be altered by anthropogenic pressures, such as the input of domestic sewage, urban storm water runoff, changes in land use, and reduced flows because of diversions (Araújo et al., 2021), which overwhelm the river’s ability to dilute pollutants (Sant’Ana & Cunha, 2022). In addition, rainy and dry periods influence the volume of water, the dilution or concentration of nutrients and contaminants, and the mobilization of sediments in the basin (Martins et al., 2021; Silva & Schwingel, 2021).

The seasonal distribution of precipitation, with more intense periods between December and May and dry periods between June and November (Table S5), also affects water quality and the ecological response of the system and aggravates the impacts of anthropogenic pressures (Liu et al., 2024; Silva & Schwingel, 2021). The spatial and temporal patterns described here were based on all sampled environmental variables, without accounting for their potential multicollinearity (see Table S6 for variable correlations and correlations with PCoA axes). Future studies aiming to use these descriptors in predictive models should consider both theoretical relevance and statistical independence among variables.

Seasonality also can influence species dynamics, such as that of *U. arrecta*, whose abundance in certain areas could be affected by seasonal variables like precipitation (Galvanese et al., 2022), resulting from changing habitat suitability, competitive interactions, dispersal, and nutrient availability along the river (Weng et al., 2020). However, the test conducted on the dominance of *U. arrecta* was insignificant (*P* > 0.13) relative to temporal and spatial variables. The environmental factors that influence the growth and establishment of *U. arrecta* are more related to nutrient availability, water quality, eutrophication, and physical habitat disturbance (Lindholm et al., 2020). This result contrasts with the study by Bora et al. (2020), which emphasized the role of salinity tolerance in facilitating the invasion of *U. arrecta* in aquatic ecosystems. We believe it is important to conduct long-term studies to monitor this coastal ecosystem to better understand the synergistic effects of abiotic disturbances and biological invasions.

**Fig. 5:**
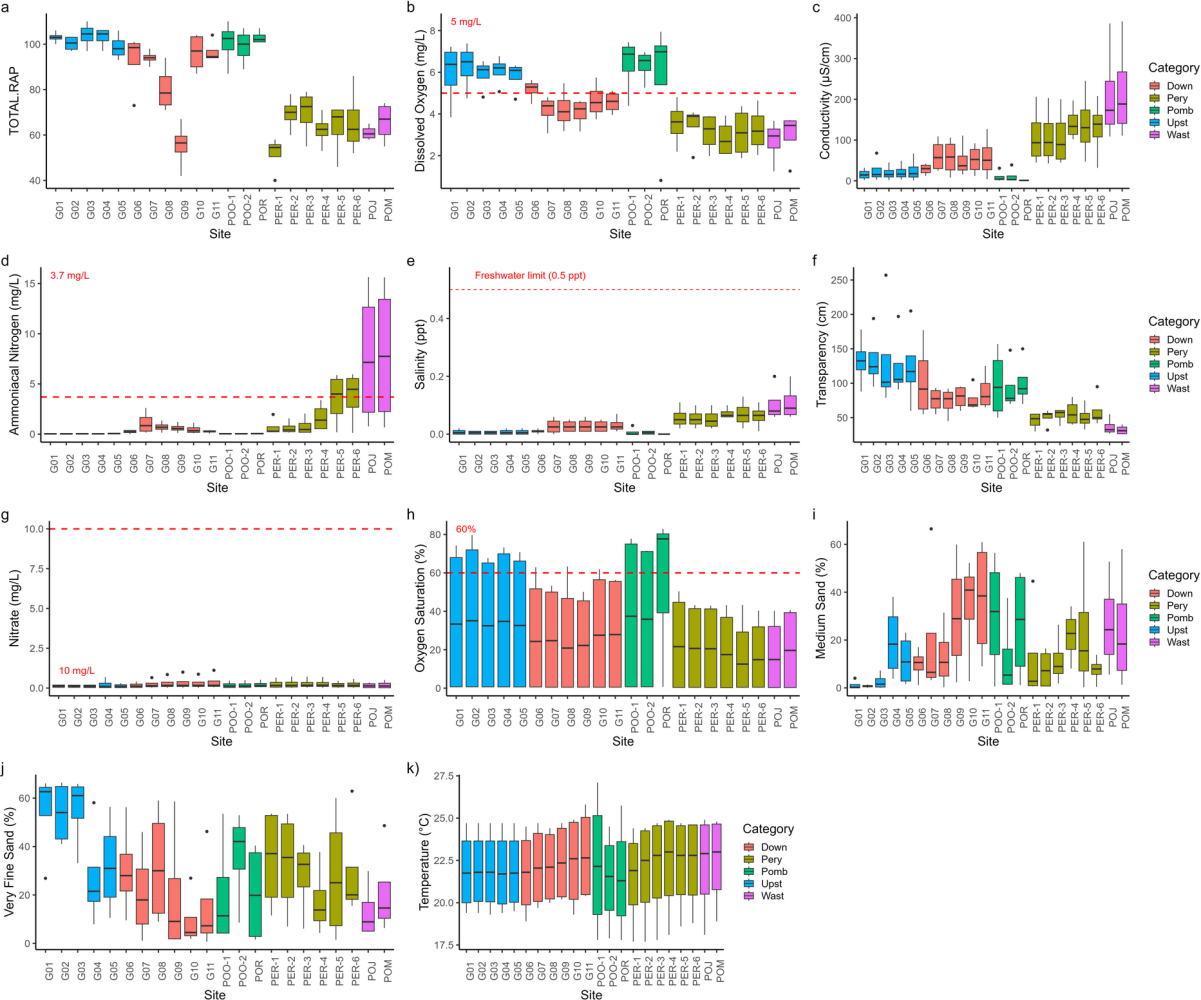
Abiotic variables driving the environmental gradient: **a** rapid assessment protocol (RAP); **b** dissolved oxygen; **c** conductivity; **d** ammoniacal nitrogen; **e** salinity; **f** transparency; **g** nitrate; **h** oxygen saturation (%); **i** medium sand; **j** very fine sand; **k** temperature. The horizontal line inside the box represents the median of the data. The lower and upper ends of the box correspond to the first (Q1) and third (Q3) quartiles, respectively, indicating the interquartile range (IQR). The “whiskers” (vertical lines extending from the box) show the range of data outside the interquartile range, typically up to 1.5 times the IQR from the quartiles. Points outside the whiskers represent outliers. The red dotted line represents the threshold concentration values established by Brazilian legislation (CONAMA Resolution No. 357/2005). Down = downstream; Pery = Pery River; Pomb = Pombas River; Upst = Upstream; Wast = wastewater disposal region

In general, the downstream Guaraguaçu sites (G7–G11) had higher conductivity (Fig. 5c), salinity (Fig. 5e), and nutrients (Fig. 5d). On the other hand, upstream Guaraguaçu sites (G1–G5) had higher dissolved oxygen levels (Fig. 5b, h). Elevated nitrate concentrations were detected in the Pery River sites (PER1–6, POJ, POM) and downstream Guaraguaçu sites, particularly at site G6, located near the confluence of the Pery and Guaraguaçu Rivers. Elevated concentrations of nitrogen in aquatic systems lead to eutrophication and contribute to the degradation of coastal ecosystems (Conley et al., 2009; Esteves, 1998). This process often results in decreased dissolved oxygen levels and deterioration of habitat quality, negatively affecting aquatic biodiversity and ecosystem functioning (Li et al., 2022; Liu et al., 2019; Rose et al., 2019). The Pery River sites likely exhibit higher pollutant loads because of the large organic load from the artificial canal system and sewage treatment plant effluents (Elste, 2021, 2019; Araújo et al., 2021).

The high electrical conductivity values (Fig. 5c) are closely associated with the concentration of dissolved salts in the water, a condition often linked to anthropogenic activities such as the discharge of industrial and domestic effluents (Chalupová et al., 2012; de Sousa et al., 2014; Thompson et al., 2012). Although salinity in a coastal river can be directly related to saline intrusions, the high salt concentrations observed in the Pery River sites were primarily the result of anthropogenic activities, such as the discharge of effluents and the presence of drainage channels and systems.

In the downstream Guaraguaçu sites (G6–G11), there were elevated levels of nitrate (Fig. 5g), medium sand (Fig. 5i), very fine sand (Fig. 5j), and temperature (Fig. 5k) and lower levels of oxygen saturation (Fig. 5h). Sites located in the Pery River (PER1 to PER6) were associated with even higher levels of nitrate, medium and very fine sand, and temperature and lower saturated dissolved oxygen levels, suggesting greater environmental impact in this area. Conversely, the Pomb sites (POO1, POO2, and POR) showed higher values of saturated dissolved oxygen, medium sand, and temperature, along with lower concentrations of nitrate and very fine sand, indicating comparatively better environmental conditions.

The spatial gradient of environmental conditions revealed that some sites with elevated nutrient concentrations were not located near evident sources of anthropogenic pollution. We believe that this could be related to the tidal variations, which affect flow dynamics and can redistribute nutrients across different river zones. Tidal fluctuations represent an important environmental driver within the basin, directly influencing habitat quality and the dynamics of floodplain ecosystems (Bilal et al., 2020; Regier et al., 2021; Sato et al., 2021). Such fluctuations can markedly affect abiotic conditions, as changes in water levels and flow patterns affect different river segments in distinct ways (Bilal et al., 2020; Indivero et al., 2021). For instance, tides and diurnal cycles often dominate the chemical variability of the water, affecting parameters such as pH and dissolved oxygen (Indivero et al., 2021). The magnitude of these influences varies according to the size and morphological structure of the river, from upstream areas to the river mouth, including tributaries (Sawakuchi et al., 2017). Consequently, fluctuations in water levels, particularly those associated with semi-diurnal floodplain inundation, introduce additional variability in abiotic parameters from hydrodynamic processes (Baumann & Smith, 2017; Ward et al., 2018). This reinforces the role of tidal influence, especially variations in water level, as a critical factor shaping environmental conditions (Baumann & Smith, 2017; Ward et al., 2018). In the case of the Guaraguaçu River, the sites G6–G11 are the most influenced by tidal action (Sant’Ana & Cunha, 2022). Therefore, the interaction between tidal fluctuations and abiotic factors highlights the complexity of tidal processes and their impact on environmental dynamics. Understanding these interactions is essential for the sustainable management of coastal aquatic ecosystems, where tidal dynamics play a crucial role in maintaining habitat and water quality.

Estuarine and coastal marine ecosystems are more vulnerable to the combined effects of multiple anthropogenic drivers of change than open ocean ecosystems (Kennish, 2021). In this study, we evaluated the environmental conditions of the Guaraguaçu River, taking into account multiple anthropogenic stressors, and found that water pollution was the primary factor driving the deterioration of environmental conditions (Table S6). However, it is important to recognize that ecosystems generally respond to multiple interacting threats, which vary in complexity depending on the characteristics of the natural environment (Lima et al., 2023).

In recent years, there has been increasing concern regarding pollution stress, particularly from land-based sources transported to coastal zones by rivers (Lu et al., 2022). This concern is reflected in one of the key global targets for achieving sustainable development in coastal systems, the protection of marine ecosystems outlined in Sustainable Development Goal 14 (United Nations, 2025). This combination of natural complexity from tidal fluctuations, combined with pollution stress and other interacting drivers, poses substantial challenges for researchers, managers, and policymakers, highlighting the need for a structured framework to guide adaptive management, project monitoring, and effective policy implementation (Lima et al., 2023).

Coastal systems subjected to anthropogenic pressures, such as those observed in the Guaraguaçu River basin, commonly exhibit environmental degradation linked to urban expansion, land use changes, and pollution. In northeastern Brazil, for instance, elevated nutrient concentrations and reduced dissolved oxygen levels in 12 tropical coastal rivers have been associated with domestic and industrial wastewater inputs (Noriega et al., 2021). In the Pearl River estuary, China, seasonal inputs of nutrients, such as phosphorus and nitrogen, have been linked to the intensification of eutrophication processes, highlighting the role of interactions between human activities and natural seasonal variability in shaping environmental conditions (Niu et al., 2020). Seasonality variation in the Guaraguaçu River basin contributed to spatial differences in environmental conditions across the river. In Cartagena Bay, Colombia, seasonal fluctuations in physical and chemical parameters and dissolved oxygen levels were evidenced, in which hypoxic conditions during the wet season were linked to vertical stratification and altered water circulation patterns (Tosic et al., 2019). These examples collectively underscore pollution as a serious source of impacts and the importance of considering temporal variability, particularly seasonality, in the monitoring and managing of coastal basins under multiple stressors.

The outcomes of this study offer valuable insights for improving environmental management in coastal river systems, particularly in regions of high ecological and economic importance such as the Guaraguaçu River basin. This basin supports critical ecosystem services and plays a strategic role in national and international markets due to its proximity to port infrastructure linked to agribusiness. The contrasting chemical and physical conditions observed along the gradient of environmental conditions emphasize the need to tailor management actions according to site-specific characteristics. In areas with better ecological status, conservation efforts should focus on preserving current conditions and preventing the introduction of new stressors. For example, expanding the extent of protected areas in the region may help maintain ecosystem integrity.

Conversely, in regions exhibiting signs of degradation, particularly those influenced by wastewater discharge and urban runoff, more intensive measures are required, such as expanding sewage infrastructure, reducing nutrient inputs, and avoiding implementing drainage projects that may further alter river conditions. These measures are essential to maintain the ecosystem services provided by this basin. The seasonal patterns identified reinforce the importance of incorporating temporal dynamics into monitoring programs to enhance early detection of environmental changes. Thus, we recommend that future research could assess long-term ecological responses and evaluate the effectiveness of mitigation actions, strengthening the scientific foundation for sustaining the integrity and functioning of tropical coastal river ecosystems.

## Conclusion

We evaluated the environmental conditions of the Guaraguaçu River by integrating multiple indicators, including rapid assessment protocols (RAP), physicochemical parameters of water and sediment, and land use patterns. The results revealed a gradient of environmental conditions that varied seasonally across different river zones. The most degraded sites were located in the Pery River tributary, an area subjected to significant pollution pressures, particularly from wastewater discharge. Water pollution emerged as the primary driver of environmental degradation in the middle and lower basin sector of the Guaraguaçu River. Notably, hydrological alterations in the tributary, implemented to regulate flow and enhance effluent dilution, have disrupted the natural continuity of the basin’s processes. This anthropogenic intervention acts as a critical breakpoint in the river’s environmental gradient, intensifying localized impacts and triggering cascading environmental effects downstream. These findings underscore the need for targeted management actions to rehabilitate and protect affected sites, which are essential for maintaining the ecological health of both aquatic and coastal ecosystems. Preserving these areas is fundamental to ensuring the environmental health of the watercourse and its capacity to support biodiversity and ecosystem services. It is crucial that environmental managers and decision-makers consider the outcomes of this assessment to prevent cumulative anthropogenic pressures from compromising the sustainability of the Guaraguaçu River and its multiple uses.

## Supplementary Information

Below is the link to the electronic supplementary material.

ESM1 (DOCX 2.71 MB)

## Data Availability

The datasets generated during and/or analyzed during the current study are available from the first author.
