# Supplementary material for: Water pollution drives environmental degradation in a seasonally influenced Neotropical coastal river
Source: Environ Monit Assess. 2026 Mar 16;198(4):322. doi: 10.1007/s10661-025-14956-w (PMC12992489; doi:10.1007/s10661-025-14956-w)
Supplement: Supplementary file 1 — (DOCX 2.71 MB) [file 10661_2025_14956_MOESM1_ESM.docx]

**Water pollution drives environmental degradation in a seasonally influenced Neotropical coastal river**

Electronic supplementary material (ESM)

**
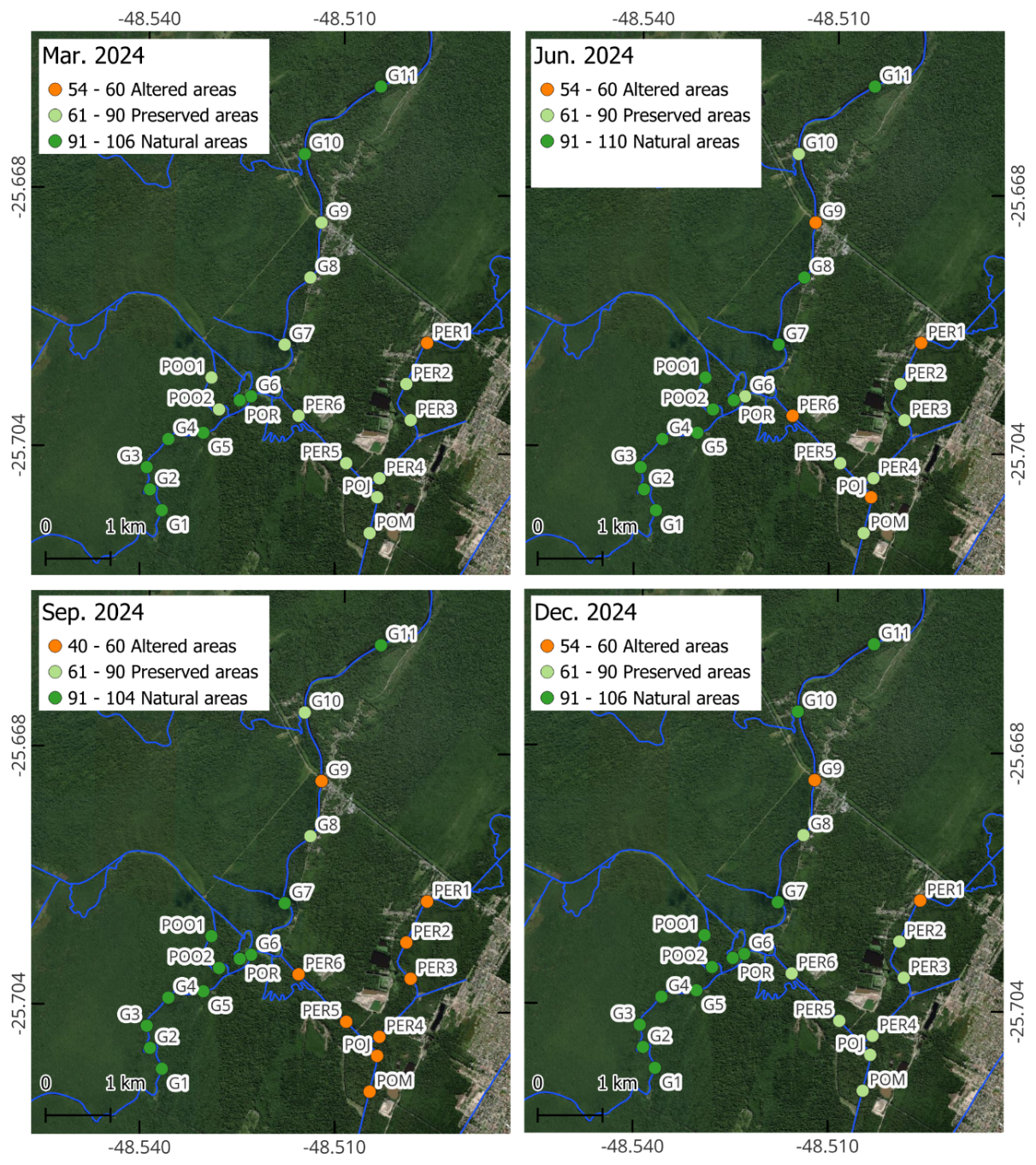
**

**Fig. S1** Environmental conditions of the sampling sites based on the average scores from the Rapid Assessment Protocol.

**Table S1** Sampling site locations

| **Sites** | **Longitude** | **Latitude** | **River zone** | **Local** |
| --- | --- | --- | --- | --- |
| **G1** | 747159,886 | 7153822,867 | Upst | Guaraguaçu River |
| **G2** | 746976,523 | 7154145,07 | Upst | Guaraguaçu River |
| **G3** | 746926,872 | 7154487,724 | Upst | Guaraguaçu River |
| **G4** | 747260,843 | 7154922,198 | Upst | Guaraguaçu River |
| **G5** | 747801,639 | 7155020,061 | Upst | Guaraguaçu River |
| **G6** | 748540,06 | 7155582,833 | Down | Guaraguaçu River |
| **G7** | 749058,81 | 7156381,476 | Down | Guaraguaçu River |
| **G8** | 749455,228 | 7157417,389 | Down | Guaraguaçu River |
| **G9** | 749628,723 | 7158267,624 | Down | Guaraguaçu River |
| **G10** | 749367,869 | 7159326,754 | Down | Guaraguaçu River |
| **G11** | 750546,077 | 7160367,232 | Down | Guaraguaçu River |
| **PER1** | 751258,616 | 7156406,914 | Pery | Pery River |
| **PER2** | 750937,384 | 7155773,644 | Pery | Pery River |
| **PER3** | 751003,208 | 7155213,845 | Pery | Pery River |
| **PER4** | 750521,769 | 7154316,582 | Pery | Pery River |
| **PER5** | 750008,583 | 7154548,628 | Pery | Pery River |
| **PER6** | 749273,034 | 7155281,534 | Pery | Pery River |
| **POJ** | 750485,671 | 7154022,083 | Wast | Pery River |
| **POM** | 750370,171 | 7153468,606 | Wast | Pery River |
| **POO1** | 747927,046 | 7155872,526 | Pombas | Pombas River |
| **POO2** | 748043,561 | 7155379,759 | Pombas | Pombas River |
| **POR** | 748364,447 | 7155520,728 | Pombas | Pombas River |

Upst= Upstream; Down= downstream; Pery= Pery River; Wast= Wastewater disposal region; Pombas= Pombas River.

**Table S2a** Rapid Assessment Protocol (adapted from Callisto et al. 2002)

| DESCRIPTION OF THE ENVIRONMENT | | | |
| --- | --- | --- | --- |
| **Location:** | | | |
| **Data:** Sampling time**:** | | | |
| Weather (situation of the day): | | | |
| Environment Type: Stream ( ) River ( ) | | | |
| Average width: | | | |
| Average depth: | | | |
| Water temperature: | | | |
| Variables | **Scoring**  **7 3 0** | | |
|  |  |  |  |
| 1. Type of occupation of the margins of the body of water (main activity) | Natural vegetation | Pasture/Agriculture | Residential occupation |
| 2. Riverbank stability | Stable banks | 30 to 60% of the riverbank is eroded. | > 60% of the bank eroded. |
| 3. Siltation | Absence of silting | Moderate siltation | Heavy siltation |
| 4. Anthropogenic disturbances | Absent | Visual domestic disturbances (presence of sewage and solid waste) | Disturbances of industrial/urban origin (factories, canalization, straightening of the river course, mining activity, waste disposal or landfills) |
| 5. Water odor | Absent | Sewage (rotten egg), characteristic of domestic sewage, organic | Oil/industrial odor |
| 6. Oiliness of water | Absent | Weak oil stains | Abundant oil stains |
| 7. Odor of sediment | Absent | Sewage (rotten egg), characteristic of domestic sewage, organic | Oil/industrial odor |
| 8. Riverbed type | Sand | Mud/silt/clay | Concrete riverbed |

**Table S2b** Rapid Assessment Protocol used in the study adapted from Callisto et al. (2002)

| **Variables** | 9 6 3 0 | | | |
| --- | --- | --- | --- | --- |
| 9. Changes in the river channel | Channeling (rectification) or dredging absent or minimal; river with normal pattern, less than 30% in changes | Some river channelization present, usually near bridge construction, between 60% and 30% | Some modification present on both banks, 90% to 60% of river modified | Modified banks over 90% of the river modified |
| 10. Presence of native riparian vegetation | Over 90% with native riparian vegetation, including trees, shrubs or macrophytes, minimal evidence of deforestation | Between 60% and 90% with native riparian vegetation; evidence of deforestation but not affecting all vegetation. | Between 30% and 60% of native riparian vegetation; evident deforestation in areas with exposed soil or cleared vegetation. | Less than 30% of native riparian forest; very pronounced deforestation |
| 11. Extent of riparian forest | Width of riparian vegetation greater than 18 m; without influence of human activities (agriculture, roads, etc.) | Width of riparian vegetation between 12 and 18 m; minimal human influence | Width of riparian vegetation between 6 and 12 m; evident human influence | Width of riparian vegetation less than 6 m; vegetation restricted or absent due to human activity |
| 12. Presence of aquatic plants | Small aquatic macrophytes and/or mosses distributed throughout the riverbed | Aquatic macrophytes and filamentous algae or mosses distributed in the river, substrate with periphyton | Filamentous algae or macrophytes on a few rocks or in some backwaters, abundant periphyton and biofilm | Absence of aquatic vegetation in the riverbed or large banks of macrophytes |
| 13. Presence of invasive aquatic plants | < 30% | 30-60% | 60-90% | > 90% |
| 14. Presence of aquatic animals | Over 90% of the number of animals (fish, aquatic insects, birds) | Between 90% and 60% of the number of animals (fish, aquatic insects, birds) | Between 60% and 30% of the number of animals (fish, aquatic insects, birds) | Between 30% of the quantity of animals (fish, aquatic insects, birds) |

**Table S3** Dominance of invasive *Urochloa arrecta* at sampling sites by season

| **Site** | **Sampling period** | ***Urochloa arrecta*** |
| --- | --- | --- |
| G1 | mar/24 | no |
| G2 | mar/24 | yes |
| G3 | mar/24 | yes |
| G4 | mar/24 | yes |
| G5 | mar/24 | no |
| G6 | mar/24 | no |
| G7 | mar/24 | yes |
| G8 | mar/24 | no |
| G9 | mar/24 | yes |
| G10 | mar/24 | no |
| G11 | mar/24 | yes |
| POO-1 | mar/24 | yes |
| POO-2 | mar/24 | yes |
| POR | mar/24 | yes |
| PER-1 | mar/24 | yes |
| PER-2 | mar/24 | yes |
| PER-3 | mar/24 | no |
| PER-4 | mar/24 | no |
| PER-5 | mar/24 | yes |
| PER-6 | mar/24 | yes |
| POJ | mar/24 | yes |
| POM | mar/24 | yes |
| G1 | jun/24 | no |
| G2 | jun/24 | yes |
| G3 | jun/24 | yes |
| G4 | jun/24 | yes |
| G5 | jun/24 | yes |
| G6 | jun/24 | no |
| G7 | jun/24 | yes |
| G8 | jun/24 | yes |
| G9 | jun/24 | yes |
| G10 | jun/24 | yes |
| G11 | jun/24 | yes |
| POO-1 | jun/24 | yes |
| POO-2 | jun/24 | yes |
| POR | jun/24 | yes |
| PER-1 | jun/24 | yes |
| PER-2 | jun/24 | yes |
| PER-3 | jun/24 | yes |
| PER-4 | jun/24 | yes |
| PER-5 | jun/24 | yes |
| PER-6 | jun/24 | yes |
| POJ | jun/24 | yes |
| POM | jun/24 | no |
| G1 | sep/24 | no |
| G2 | sep/24 | yes |
| G3 | sep/24 | yes |
| G4 | sep/24 | yes |
| G5 | sep/24 | no |
| G6 | sep/24 | yes |
| G7 | sep/24 | yes |
| G8 | sep/24 | yes |
| G9 | sep/24 | yes |
| G10 | sep/24 | yes |
| G11 | sep/24 | yes |
| POO-1 | sep/24 | yes |
| POO-2 | sep/24 | yes |
| POR | sep/24 | no |
| PER-1 | sep/24 | yes |
| PER-2 | sep/24 | yes |
| PER-3 | sep/24 | yes |
| PER-4 | sep/24 | yes |
| PER-5 | sep/24 | yes |
| PER-6 | sep/24 | yes |
| POJ | sep/24 | yes |
| POM | sep/24 | no |
| G1 | dec/24 | no |
| G2 | dec/24 | yes |
| G3 | dec/24 | no |
| G4 | dec/24 | yes |
| G5 | dec/24 | yes |
| G6 | dec/24 | no |
| G7 | dec/24 | yes |
| G8 | dec/24 | no |
| G9 | dec/24 | yes |
| G10 | dec/24 | no |
| G11 | dec/24 | yes |
| POO-1 | dec/24 | yes |
| POO-2 | dec/24 | yes |
| POR | dec/24 | yes |
| PER-1 | dec/24 | yes |
| PER-2 | dec/24 | yes |
| PER-3 | dec/24 | yes |
| PER-4 | dec/24 | yes |
| PER-5 | dec/24 | no |
| PER-6 | dec/24 | yes |
| POJ | dec/24 | yes |
| POM | dec/24 | no |

Mar = March/2024; Jun = June/2024; Sep = September/2024; Dez = December/2024

**Table S4** Means and standard deviation of measured abiotic variables

| **Site** | **Class** | **RAP** | **OM%** | **Tem.** | **pH** | **DO** | **Cond** | **Sali.** | **TDS** | **Nit.** | **N-amm.** | **Sec. (cm)** | **V. C. Sand (%)** | **C. Sand (%)** |
| --- | --- | --- | --- | --- | --- | --- | --- | --- | --- | --- | --- | --- | --- | --- |
| **G1** | Upst | 103±2,50 | 1,29±2,21 | 21,9±2,507 | 7,102±0,90 | 5,96±1,52 | 15,75±13,12 | 0,008±0,01 | 4,76±4,64 | 0,11±0,09 | 0,04±0,04 | 13,75±26,39 | 0±0 | 0±0 |
| **G2** | Upst | 100±3,20 | 1,33±4,28 | 21,92±2,5 | 6,932±1,0 | 6,29±1,13 | 25,5±29,1 | 0,01±0,01 | 4,50±5,06 | 0,11±0,08 | 0,04±0,04 | 13,25±40,6 | 0±0 | 0,19±0,38 |
| **G3** | Upst | 104±5,47 | 1,43±3,44 | 21,91±2,5 | 6,727±1,2 | 5,89±0,75 | 19,5±18,4 | 0,01±0,01 | 4,76±5,18 | 0,11±0,09 | 0,04±0,05 | 13,75±89,8 | 0±0 | 0,145±0,2 |
| **G4** | Upst | 103±4,24 | 1,18±6,62 | 21,87±2,6 | 6,757±0,9 | 6,06±0,72 | 21,3±19,9 | 0,01±0,01 | 4,76±5,49 | 0,22±0,31 | 0,04±0,04 | 12,75±52,8 | 0±0 | 2,8±3,33 |
| **G5** | Upst | 98,8±5,61 | 1,53±15,4 | 21,92±2,5 | 6,872±0,9 | 5,80±0,74 | 25,8±28,9 | 0,01±0,01 | 5,76±6,89 | 0,13±0,13 | 0,06±0,05 | 12,75±50,8 | 0,12±0,24 | 2,547±3,7 |
| **G6** | Down | 92,8±12,8 | 0,78±1,33 | 21,76±2,6 | 6,937±1,4 | 5,17±0,49 | 28,5±13,6 | 0,01±0,01 | 10,00±9,05 | 0,16±0,17 | 0,23±0,17 | 10,75±56,0 | 0±0 | 0,155±0,3 |
| **G7** | Down | 94±3,26 | 2,7±13,4 | 22,12±2,5 | 7,417±0,8 | 4,16±0,77 | 62±40,32 | 0,03±0,03 | 30,25±27,2 | 0,24±0,28 | 1,11±1,12 | 75,75±13,8 | 0,75±1,50 | 3,417±6,8 |
| **G8** | Down | 80,5±10,3 | 1,12±3,29 | 22,16±2,3 | 7,11±0,84 | 4,21±0,97 | 57,8±45,1 | 0,03±0,02 | 30,3±27,0 | 0,30±0,37 | 0,72±0,49 | 73±11,2 | 0,03±0,06 | 1,59±2,03 |
| **G9** | Down | 55,5±10,3 | 0,26±0,45 | 22,41±2,4 | 7,075±0,9 | 4,06±0,66 | 50,25±42,1 | 0,03±0,03 | 38,75±37,3 | 0,34±0,44 | 0,61±0,45 | 79,5±19,7 | 1,53±1,98 | 9,95±10,96 |
| **G10** | Down | 96,3±8,53 | 0,29±0,84 | 22,35±2,9 | 6,827±0,9 | 4,65±0,87 | 52,25±36,3 | 0,03±0,02 | 27,00±23,4 | 0,30±0,38 | 0,47±0,46 | 76,75±21,1 | 0±0 | 8,19±11,9 |
| **G11** | Down | 96,8±4,9 | 0,61±1,8 | 22,9±2,8 | 7,23±0,5 | 4,57±0,5 | 58±52,5 | 0,03±0,03 | 30,8±30,0 | 0,37±0,5 | 0,25±0,2 | 87,8±27,8 | 0±0 | 2,40±1,93 |
| **POO-1** | Pomb | 101±9,746 | 1,80±11,9 | 22,29±4,3 | 6,042±0,5 | 6,39±1,38 | 10±14,07 | 0,008±0,02 | 3,01±4,08 | 0,18±0,20 | 0,05±0,05 | 98,75±54,9 | 2,84±3,17 | 11,24±6,8 |
| **POO-2** | Pomb | 99±7,831 | 3,08±14,0 | 21,35±2,9 | 6,37±0,4 | 6,34±0,76 | 11,75±18,2 | 0,01±0,01 | 1,01±0,81 | 0,18±0,21 | 0,05±0,05 | 93,5±43,4 | 0,92±1,84 | 1,97±3,74 |
| **POR** | Pomb | 103±2,82 | 0,71±1,61 | 21,5±2,57 | 6,515±0,92 | 5,68±0,55 | 2±1,15 | 0±0 | 1,5±0,56 | 0,22±0,21 | 0,057±0,056 | 101±33,6 | 0±0 | 4,625±8,13 |
| **PER-1** | Pery | 51,8±8,01 | 1,41±3,29 | 21,5±2,9 | 7,12±1,32 | 3,56±1,09 | 109,5±71,87 | 0,057±0,038 | 53,3±47,3 | 0,25±0,28 | 0,68±0,85 | 45,5±14,4 | 0,09±0,17 | 0,905±1,72 |
| **PER-2** | Pery | 69,5±7,41 | 2,01±10,6 | 21,8±3,2 | 7,407±0,78 | 3,44±1,02 | 108,5±70,69 | 0,055±0,034 | 52,8±45,78 | 0,26±0,31 | 0,64±0,63 | 50,5±2,9 | 0±0 | 0,023±0,05 |
| **PER-3** | Pery | 69,8±10,68 | 0,92±1,13 | 22,02±3,3 | 7,65±0,99 | 3,11±0,93 | 105,8±71,44 | 0,052±0,035 | 50,5±45,9 | 0,27±0,30 | 0,79±0,87 | 53,5±3,2 | 0±0 | 0,267±0,32 |
| **PER-4** | Pery | 62,3±7,36 | 0,46±0,65 | 22,3±3,3 | 7,562±0,69 | 2,84±0,90 | 141,5±41,82 | 0,072±0,018 | 56,3±43,4 | 0,26±0,29 | 1,67±1,39 | 57±18,6 | 0±0 | 1,277±0,70 |
| **PER-5** | Pery | 63,3±11,84 | 0,40±0,44 | 22,2±2,9 | 6,982±0,53 | 3,11±1,22 | 138,3±82,87 | 0,07±0,046 | 69,01±55,18 | 0,20±0,19 | 3,51±2,61 | 50,75±21,1 | 0,02±0,04 | 1,92±2,28 |
| **PER-6** | Pery | 65,8±14,66 | 0,35±0,098 | 22,24±2,9 | 7,747±1,33 | 3,25±1,14 | 129,5±72,85 | 0,062±0,041 | 65,50±47,3 | 0,23±0,24 | 3,75±2,63 | 59,25±28,6 | 0±0 | 0,19±0,38 |
| **POJ** | Wast | 103±2,82 | 0,52±1,03 | 22,20±3,16 | 8,042±0,84 | 2,7±1,04 | 210,3±122,56 | 0,10±0,064 | 99,02±85,22 | 0,15±0,16 | 7,68±7,12 | 36,75±14,4 | 0±0 | 0,68±0,65 |
| **POM** | Wast | 65,8±8,88 | 1,15±3,95 | 22,4±2,82 | 8,167±1,09 | 2,96±1,16 | 219,8±12,71 | 0,11±0,063 | 97,02±88,6 | 0,19±0,23 | 7,94±7,32 | 32±7,09 | 0±0 | 1,572±1,85 |

**Upst**= Upstream, **Down**= downstream, **Pomb**= Pombas River, **Pery**= Pery River, **Wast**= Wastewater disposal region. **RAP**= rapid assessment protocol, **OM%**= organic matter, **Temp**= temperature **°C**, **pH**= potential hydrogen, **DO**= dissolved oxygen**(mgL-1)**, **Cond**= conductivity**(µs/cm2)**, **Sali**= salinity, **TDS**= total dissolved solids**(mgL-1)**, **Nit**= nitrate**(mgL-1)**, **N-amm**= ammoniacal nitrogen**(mgL-1)**, **Sec**= Secchi**(cm)**, **V.C. Sand**= very coarse sand**(%)**, **C. Sand**= coarse sand**(%)**

**Table S4** Means and standard deviation of measured abiotic variables **Continued**

| **Site** | **Class** | **Pot.Red** | **DO**  **(sat%)** | **Turb.** | **Nitrit** | **Total-P** | **Ortho-P** | **Nat. Veg.**  **(%)** | **Past (%)** | **Urban %)** | **M. Sand (%)** | **F. Sand (%)** | **V. F. Sand (%)** | **Mud (%)** |
| --- | --- | --- | --- | --- | --- | --- | --- | --- | --- | --- | --- | --- | --- | --- |
| **G1** | Upst | 6,95±97,9 | 35,3±40,2 | 15,3±25,03 | 0,006±0,008 | 0,26±0,23 | 0,015±0,034 | 100±0 | 0±0 | 0±0 | 1,14±1,9 | 27,6±10,1 | 54,6±18,6 | 16,6±10,6 |
| **G2** | Upst | 16,9±91,0 | 37,6±42,9 | 5,00±3,18 | 0,006±0,008 | 0,095±0,05 | 0,017±0,028 | 100±0 | 0±0 | 0±0 | 0,72±0,5 | 34,2±10,1 | 53,9±13,3 | 10,9±6,1 |
| **G3** | Upst | 16,8±91,3 | 33,3±37,7 | 5,03±4,45 | 0,006±0,008 | 0,27±0,25 | 0,25±0,04 | 100±0 | 0±0 | 0±0 | 2,62±3,3 | 29,6±14,0 | 55,3±15,2 | 12,3±3,6 |
| **G4** | Upst | 11,7±71,8 | 35,8±40,6 | 15,4±14,9 | 0,006±0,008 | 0,22±0,15 | 0,15±0,02 | 100±0 | 0±0 | 0±0 | 19,5±15,7 | 44,4±18,9 | 27,3±21,6 | 5,85±7,9 |
| **G5** | Upst | 1,48±76,5 | 34,2±38,8 | 16,9±15,7 | 0,006±0,009 | 0,40±0,33 | 0,33±0,04 | 100±0 | 0±0 | 0±0 | 11,6±10,7 | 47,4±11,6 | 32,2±20,14 | 6,04±9,4 |
| **G6** | Down | -20,6±59,5 | 28,0±32,5 | 4,35±2,67 | 0,008±0,012 | 0,16±0,08 | 0,08±0,04 | 100±0 | 0±0 | 0±0 | 9,85±6,57 | 57,5±16,0 | 30,5±19,4 | 1,96±1,5 |
| **G7** | Down | -40,1±69,9 | 25,8±29,4 | 6,33±2,28 | 0,016±0,018 | 0,24±0,14 | 0,14±0,12 | 99±0 | 01±0 | 0±0 | 20,6±30,5 | 46,7±33,3 | 20,7±19,6 | 7,68±14,6 |
| **G8** | Down | -15,4±62,1 | 26,3±31,9 | 5,24±0,77 | 0,018±0,019 | 0,26±0,16 | 0,16±0,08 | 87±0 | 13±0 | 0±0 | 13,2±13,5 | 43,5±21,7 | 32±24,5 | 9,58±13,8 |
| **G9** | Down | -17,8±62,6 | 23,7±27,1 | 4,99±0,78 | 0,019±0,019 | 0,27±0,27 | 0,27±0,10 | 55±0 | 4±0 | 41±0 | 30,04±25,3 | 36,0±21,1 | 19,6±26,9 | 2,57±3,7 |
| **G10** | Down | 39,9±109,5 | 29,4±33,6 | 4,49±0,80 | 0,018±0,017 | 0,26±0,21 | 0,21±0,07 | 94±0 | 06±0 | 0±0 | 34,1±21,8 | 46,2±15,4 | 9,5±11,7 | 1,89±3,7 |
| **G11** | Down | -1,95±76,4 | 28,1±31,9 | 4,36±0,92 | 0,019±0,017 | 0,30±0,22 | 0,22±0,096 | 100±0 | 0±0 | 0±0 | 36,7±25,2 | 44,8±17,8 | 15,4±20,8 | 0,63±1,14 |
| **POO-1** | Pomb | 38,2±81,01 | 38,3±43,4 | 8,38±8,85 | 0,007±0,010 | 0,17±0,13 | 0,13±0,03 | 100±0 | 0±0 | 0±0 | 30,2±25,3 | 26,1±10,7 | 20,13±23,3 | 9,91±11,4 |
| **POO-2** | Pomb | 29,9±88,20 | 35,9±40,7 | 23,7±21,5 | 0,007±0,010 | 0,095±0,07 | 0,07±0,13 | 100± | 0±0 | 0±0 | 12,3±17,1 | 28,8±9,2 | 36,42±19,5 | 19,4±16,7 |
| **POR** | Pomb | -34,2±15,12 | 40,5±46,0 | 8,98±3,95 | 0,008±0,011 | 0,08±0,04 | 0,04±0,03 | 100±0 | 0±0 | 0±0 | 26,5±23,6 | 44,8±9,9 | 20,4±20,9 | 3,53±4,52 |
| **PER-1** | Pery | -37,4±50,23 | 23,5±27,0 | 18,5±11,4 | 0,015±0,015 | 0,16±0,03 | 0,03±0,07 | 63,5±0 | 02±0 | 34,5±0 | 12,6±21,3 | 48,3±13,8 | 34,8±21,5 | 3,13±2,32 |
| **PER-2** | Pery | -34,8±66,2 | 21,1±24,1 | 17,9±13,5 | 0,016±0,016 | 0,19±0,13 | 0,13±0,08 | 86±0 | 14±0 | 0±0 | 7,86±8,32 | 46,9±27,3 | 32,9±21,8 | 12,3±13,7 |
| **PER-3** | Pery | -55,2±71,3 | 21,0±24,0 | 20,8±24,5 | 0,018±0,020 | 0,27±0,11 | 0,11±0,09 | 100±0 | 0±0 | 0±0 | 11,5±10,5 | 58,1±8,0 | 28±15,4 | 1,99±1,76 |
| **PER-4** | Pery | -42,2±58,7 | 19,6±22,8 | 19,4±18,4 | 0,019±0,021 | 0,27±0,06 | 0,06±0,15 | 91±0 | 09±0 | 0±0 | 21,9±11,1 | 57,8±8,1 | 17,4±14,4 | 1,47±2,27 |
| **PER-5** | Pery | -23,8±71,3 | 17,1±21,6 | 15,3±7,98 | 0,018±0,022 | 0,44±0,27 | 0,27±0,26 | 92±0 | 08±0 | 0±0 | 23,09±26,8 | 41,2±14,9 | 27,9±27,4 | 5,84±5,79 |
| **PER-6** | Pery | -51,6±80,02 | 17,5±20,68 | 16,2±9,17 | 0,019±0,023 | 0,51±0,44 | 0,44±0,28 | 88±0 | 12±0 | 0±0 | 7,52±5,49 | 59,8±21,8 | 29,6±22,3 | 2,8±4,34 |
| **POJ** | Wast | -58,7±77,8 | 17,5±20,7 | 50,2±35,69 | 0,018±0,013 | 0,80±0,,56 | 0,56±0,46 | 100±0 | 0±0 | 0±0 | 26,7±20,3 | 58,5±11,9 | 13,10±11,7 | 0,92±1,02 |
| **POM** | Wast | -59,4±78,5 | 2,00±22,8 | 29,5±26,97 | 0,017±0,013 | 0,69±0,44 | 0,44±0,46 | 100±0 | 0±0 | 0±0 | 24,01±25,1 | 49,9±18,0 | 21,1±18,9 | 3,36±5,33 |

.

**Upst**= Upstream, **Down**= downstream, **Pomb**= Pombas River, **Pery**= Pery River, **Wast**= Wastewater disposal region. **Pot.Red**= Oxidation-Reduction Potential, **DO(sat%)**=Dissolved Oxygen (sat%), **Turb**= Turbidity, **Nitrit**= Nitrite**(mgL-1)**, **Total-P**= Total Phosphorus**(mgL-1)**, **Ortho-P**= Orthophosphate**(mgL-1)**, **Nat. Veg.**= Natural vegetation**(%)**, **Urb**= Urbanization**(%)**, **Past**= Pastures**(%)**,**Urban**= Urbanization**(%),M. Sand**= medium sand**(%)**, **F. Sand**= fine sand**(%)**, **V.F. Sand**= very fine sand**(%)**

**Table S5.** Accumulated precipitation in the study region throughout the 2024 sampling period, (from AGUASPARANÁ – Paraná Water Institute, Hydrological Information System).

| **Month** | **Total accumulated precipitation for the month (millimeters)** |
| --- | --- |
| March | 205.4 |
| June | 61.2 |
| September | 57.7 |
| December | 402.1 |

Data from: http://www.sih-web.aguasparana.pr.gov.br/

**Table S6.** Values ​​of the abiotic variable axes retained in the PCoA.

| **Variables** | **Axis 1** | **Axis 2** |
| --- | --- | --- |
| Total Rapid Assessment Protocol (RAP) | 0.72576082 | -0.04537876 |
| Organic matter% | 0.23657022 | 0.48970529 |
| Temperature | 0.19835766 | -0.60333592 |
| pH | -0.59562966 | 0.45395725 |
| Dissolved Oxygen (mgL-1) | 0.76100551 | 0.22347657 |
| Conductivity (us.cm²) | -0.80180239 | -0.11894395 |
| Salinity | -0.91078197 | -0.01951752 |
| Oxidation-Reduction Potential (Pot.Redox) | 0.49697419 | -0.54277906 |
| Total Dissolved Solids (mgL-1) | -0.63427833 | 0.54800481 |
| Dissolved Oxygen (sat%) | 0.20432113 | 0.61606096 |
| Turbidity | -0.57426455 | -0.29013093 |
| N-nitrate (mgL.1) | -0.17067446 | 0.68002075 |
| N-nitrite (mgL.1) | -0.57084486 | -0.15026461 |
| N- Ammoniacal (mgL.1) | -0.86799792 | 0.03857558 |
| Total Phosphorus [Total-P (mgL-1)] | -0.63368127 | 0.19034272 |
| Orthophosphate [Ortho-P (mgL-1)] | -0.79978733 | 0.19530241 |
| Natural vegetation | 0.43257169 | 0.14580408 |
| Urbanization | -0.14944513 | -0.12742095 |
| Pastures | -0.43042940 | -0.10353572 |
| Water transparency [Secchi (cm)] | 0.55880382 | 0.36805709 |
| Very coarse sand | 0.14692258 | -0.46644231 |
| Coarse sand | 0.08592184 | -0.56461973 |
| Medium sand | -0.16062219 | -0.80876286 |
| Fine sand | -0.51152512 | -0.06236977 |
| Very fine sand | 0.32441824 | 0.65931949 |
| Mud | 0.37288324 | 0.61831292 |
